# Supplementary material for: Accuracy of ICD-9 codes in identifying patients with peptic ulcer and gastrointestinal hemorrhage in the regional healthcare administrative database of Umbria
Source: PLoS One. 2020 Jul 6;15(7):e0235714. doi: 10.1371/journal.pone.0235714 (PMC7337287; doi:10.1371/journal.pone.0235714)
Supplement: S1 Dataset — (PDF) [file pone.0235714.s002.pdf]

Gastric ulcer

Number Clinical Chart	Hospital	Gender	Patient's age on admission	Primary Diagnosis (ICD-9)	Gastroscopy positive	Other positive instrumental test	Surgery positive	Validation
Chart_1	Hospital_1	F	62	53190	0	1	0	0
Chart_2	Hospital_1	M	75	53130	1	1	0	1
Chart_3	Hospital_1	F	88	53110	2	1	0	1
Chart_4	Hospital_1	M	77	53140	1	0	0	1
Chart_5	Hospital_1	F	87	53100	1	0	0	1
Chart_6	Hospital_1	M	56	53100	1	2	0	1
Chart_7	Hospital_1	F	81	53130	1	2	0	1
Chart_8	Hospital_1	M	46	53110	1	1	0	1
Chart_9	Hospital_1	M	60	53130	1	2	0	1
Chart_10	Hospital_1	M	89	53100	1	2	0	1
Chart_11	Hospital_1	F	60	53100	1	2	0	1
Chart_12	Hospital_1	M	60	53100	1	2	0	1
Chart_13	Hospital_1	M	70	53140	1	2	0	1
Chart_14	Hospital_1	M	84	53100	1	2	0	1
Chart_15	Hospital_1	F	81	53190	1	0	0	1
Chart_16	Hospital_1	F	85	53100	1	2	0	1
Chart_17	Hospital_1	M	69	53130	1	0	0	1
Chart_18	Hospital_1	M	80	53100	1	2	0	1
Chart_19	Hospital_1	F	86	53100	1	0	0	1
Chart_20	Hospital_1	M	80	53141	1	2	0	1
Chart_21	Hospital_1	F	76	53100	1	2	0	1
Chart_22	Hospital_1	M	61	53100	1	1	0	1
Chart_23	Hospital_1	M	54	53100	1	2	1	1
Chart_24	Hospital_1	F	85	53190	1	0	0	1
Chart_25	Hospital_1	F	76	53170	1	0	0	1
Chart_26	Hospital_1	F	66	53100	0	1	0	0
Chart_27	Hospital_1	M	84	53100	1	2	0	1
Chart_28	Hospital_1	M	40	53130	1	0	0	1

Number Clinical Chart	Hospital	Gender	Patient's age on admission	Primary Diagnosis (ICD-9)	Gastroscopy positive	Other positive instrumental test	Surgery positive	Validation
Chart_29	Hospital_1	M	51	53130	0	0	0	0
Chart_30	Hospital_1	F	58	53100	1	0	0	1
Chart_31	Hospital_1	M	74	53130	1	0	0	1
Chart_32	Hospital_1	M	63	53100	1	0	0	1
Chart_33	Hospital_1	F	84	53100	1	0	0	1
Chart_34	Hospital_1	F	82	53100	1	0	0	1
Chart_35	Hospital_1	F	55	53100	1	0	0	1
Chart_36	Hospital_1	M	85	53100	1	0	0	1
Chart_37	Hospital_1	M	64	53100	1	0	0	1
Chart_38	Hospital_1	M	78	53100	1	0	0	1
Chart_39	Hospital_1	M	60	53101	1	2	0	1
Chart_40	Hospital_1	M	60	53110	2	1	0	1
Chart_41	Hospital_1	F	88	53100	1	0	0	1
Chart_42	Hospital_1	M	88	53100	1	0	0	1
Chart_43	Hospital_1	M	50	53110	2	1	0	1
Chart_44	Hospital_1	M	82	53100	1	2	0	1
Chart_45	Hospital_1	F	82	53100	1	2	0	1
Chart_46	Hospital_1	F	70	53131	1	0	0	1
Chart_47	Hospital_1	M	70	53100	0	0	0	0
Chart_48	Hospital_1	M	58	53100	1	0	0	1
Chart_49	Hospital_2	M	43	53100	1	2	0	1
Chart_50	Hospital_2	M	60	53100	1	0	0	1
Chart_51	Hospital_2	M	39	53190	1	2	0	1
Chart_52	Hospital_2	M	72	53130	1	0	0	1
Chart_53	Hospital_2	M	60	53100	1	1	0	1
Chart_54	Hospital_2	F	77	53100	0	0	0	0
Chart_55	Hospital_2	M	56	53100	1	2	0	1
Chart_56	Hospital_2	M	41	53110	n.a.	n.a.	n.a.	n.a.
Chart_57	Hospital_2	M	90	53130	1	2	0	1
Chart_58	Hospital_2	F	79	53100	1	0	0	1

Number Clinical Chart	Hospital	Gender	Patient's age on admission	Primary Diagnosis (ICD-9)	Gastroscopy positive	Other positive instrumental test	Surgery positive	Validation
Chart_59	Hospital_2	M	51	53100	1	2	1	1
Chart_60	Hospital_2	F	86	53100	1	0	0	1
Chart_61	Hospital_2	M	38	53100	0	2	0	0
Chart_62	Hospital_2	F	73	53110	2	1	0	1
Chart_63	Hospital_2	M	70	53100	1	0	0	1
Chart_64	Hospital_2	M	85	53100	1	2	0	1
Chart_65	Hospital_2	M	76	53100	1	1	0	1
Chart_66	Hospital_2	F	67	53130	1	2	0	1
Chart_67	Hospital_2	F	66	53100	1	2	1	1
Chart_68	Hospital_2	F	87	53110	2	1	1	1
Chart_69	Hospital_2	M	32	53111	2	1	1	1
Chart_70	Hospital_2	F	87	53100	1	0	0	1
Chart_71	Hospital_2	F	75	53100	n.a.	n.a.	n.a.	n.a.
Chart_72	Hospital_2	M	81	53100	1	0	0	1
Chart_73	Hospital_2	M	84	53100	0	2	0	0
Chart_74	Hospital_2	M	73	53100	1	0	0	1
Chart_75	Hospital_2	M	55	53100	1	2	0	1
Chart_76	Hospital_2	M	49	53100	1	1	0	1
Chart_77	Hospital_2	M	76	53131	1	0	0	1
Chart_78	Hospital_2	M	82	53110	2	1	1	1
Chart_79	Hospital_2	F	81	53100	1	2	0	1
Chart_80	Hospital_2	M	93	53100	1	0	0	1
Chart_81	Hospital_2	M	87	53130	1	0	0	1
Chart_82	Hospital_2	M	45	53100	1	0	0	1
Chart_83	Hospital_2	F	59	53130	1	2	0	1
Chart_84	Hospital_2	M	76	53100	1	2	0	1
Chart_85	Hospital_2	M	62	53100	1	0	0	1
Chart_86	Hospital_2	M	45	53100	0	0	0	0
Chart_87	Hospital_2	F	44	53110	1	1	1	1
Chart_88	Hospital_2	F	66	53190	1	0	0	1

Number Clinical Chart	Hospital	Gender	Patient's age on admission	Primary Diagnosis (ICD-9)	Gastroscopy positive	Other positive instrumental test	Surgery positive	Validation
Chart_89	Hospital_2	M	78	53130	1	0	0	1
Chart_90	Hospital_3	F	75	53130	1	0	0	1
Chart_91	Hospital_3	F	97	53130	1	2	0	1
Chart_92	Hospital_3	F	80	53100	1	1	1	1
Chart_93	Hospital_3	M	81	53130	1	2	0	1
Chart_94	Hospital_3	F	95	53100	1	2	0	1
Chart_95	Hospital_3	F	51	53131	1	2	0	1
Chart_96	Hospital_3	M	72	53100	1	2	0	1
Chart_97	Hospital_4	F	79	53100	1	2	0	1
Chart_98	Hospital_4	M	78	53110	2	1	1	1
Chart_99	Hospital_4	F	86	53100	1	2	0	1
Chart_100	Hospital_5	F	81	53100	1	1	0	1
Chart_101	Hospital_5	M	63	53101	1	2	0	1
Chart_102	Hospital_5	M	68	53140	1	2	1	1
Chart_103	Hospital_5	F	76	53101	1	2	0	1
Chart_104	Hospital_5	M	27	53130	1	2	0	1
Chart_105	Hospital_5	F	82	53100	1	0	0	1
Chart_106	Hospital_5	M	81	53100	1	2	0	1
Chart_107	Hospital_5	F	58	53100	1	0	0	1
Chart_108	Hospital_5	F	89	53120	1	2	1	1
Chart_109	Hospital_5	M	79	53190	0	0	0	0
Chart_110	Hospital_5	M	68	53100	1	0	0	1
Chart_111	Hospital_6	M	83	53170	1	0	0	1
Chart_112	Hospital_6	M	60	53100	1	1	0	1
Chart_113	Hospital_6	M	82	53100	1	2	0	1
Chart_114	Hospital_6	F	85	53100	0	0	0	0
Chart_115	Hospital_6	M	99	53100	1	1	0	1
Chart_116	Hospital_6	F	78	53111	1	2	1	1
Chart_117	Hospital_6	F	76	53100	1	2	0	1
Chart_118	Hospital_6	M	62	53110	2	1	1	1

Number Clinical Chart	Hospital	Gender	Patient's age on admission	Primary Diagnosis (ICD-9)	Gastroscopy positive	Other positive instrumental test	Surgery positive	Validation
Chart_119	Hospital_6	F	75	53100	1	0	0	1
Chart_120	Hospital_6	F	77	53100	1	2	0	1
Chart_121	Hospital_6	M	91	53100	0	0	0	0
Chart_122	Hospital_6	F	79	53100	1	2	0	1
Chart_123	Hospital_6	M	86	53130	1	2	0	1
Chart_124	Hospital_7	M	87	53111	1	2	0	1
Chart_125	Hospital_7	M	60	53101	1	2	0	1
Chart_126	Hospital_7	M	93	53121	1	2	0	1
Chart_127	Hospital_7	M	86	53111	1	2	0	1
Chart_128	Hospital_7	F	74	53100	1	1	0	1
Chart_129	Hospital_7	M	77	53111	1	2	1	1
Chart_130	Hospital_7	F	49	53100	1	2	0	1

Legend: 0=no; 1=yes; 2=not reported; n.a.=clinical chart not available

Duodenal ulcer

Number Clinical Chart	Hospital	Gender	Patient's age on admission	Primary Diagnosis (ICD-9)	Gastroscopy positive	Other positive instrumental test	Surgery positive	Validation
Chart_1	Hospital_1	F	58	53200	1	0	0	1
Chart_2	Hospital_1	M	72	53290	1	0	0	1
Chart_3	Hospital_1	M	55	53290	1	0	0	1
Chart_4	Hospital_1	F	51	53230	1	0	0	1
Chart_5	Hospital_1	F	84	53201	1	1	0	1
Chart_6	Hospital_1	F	79	53200	1	0	0	1
Chart_7	Hospital_1	M	36	53290	1	0	0	1
Chart_8	Hospital_1	M	74	53200	1	0	0	1
Chart_9	Hospital_1	M	77	53200	1	2	0	1
Chart_10	Hospital_1	F	83	53200	1	2	0	1
Chart_11	Hospital_1	F	83	53201	1	2	0	1
Chart_12	Hospital_1	F	79	53201	1	2	0	1
Chart_13	Hospital_1	F	82	53220	1	0	1	1
Chart_14	Hospital_1	M	50	53231	1	0	0	1
Chart_15	Hospital_1	M	56	53200	1	2	1	1
Chart_16	Hospital_1	M	58	53200	1	0	0	1
Chart_17	Hospital_1	M	42	53200	1	2	0	1
Chart_18	Hospital_1	M	34	53200	1	2	0	1
Chart_19	Hospital_1	M	81	53250	2	1	1	1
Chart_20	Hospital_1	F	73	53200	1	1	0	1
Chart_21	Hospital_1	M	75	53200	1	2	0	1
Chart_22	Hospital_1	M	42	53230	1	0	0	1
Chart_23	Hospital_1	M	75	53231	1	0	0	1
Chart_24	Hospital_1	M	88	53200	1	2	0	1
Chart_25	Hospital_1	M	46	53230	1	0	0	1
Chart_26	Hospital_1	F	53	53200	1	0	0	1
Chart_27	Hospital_1	M	42	53200	1	0	0	1
Chart_28	Hospital_1	M	84	53200	1	0	0	1

Number Clinical Chart	Hospital	Gender	Patient's age on admission	Primary Diagnosis (ICD-9)	Gastroscopy positive	Other positive instrumental test	Surgery positive	Validation
Chart_29	Hospital_1	M	72	53291	1	0	0	1
Chart_30	Hospital_1	F	84	53200	1	0	0	1
Chart_31	Hospital_1	M	86	53200	1	0	0	1
Chart_32	Hospital_1	F	57	53291	1	0	0	1
Chart_33	Hospital_1	M	58	53200	1	1	0	1
Chart_34	Hospital_1	M	39	53230	1	0	0	1
Chart_35	Hospital_1	M	57	53230	1	0	0	1
Chart_36	Hospital_1	M	58	53250	2	1	0	1
Chart_37	Hospital_1	M	64	53200	1	0	0	1
Chart_38	Hospital_1	M	83	53200	1	2	0	1
Chart_39	Hospital_2	F	77	53230	1	0	0	1
Chart_40	Hospital_2	M	47	53200	1	2	0	1
Chart_41	Hospital_2	F	82	53240	1	2	1	1
Chart_42	Hospital_2	M	58	53200	1	0	0	1
Chart_43	Hospital_2	F	85	53240	1	0	0	1
Chart_44	Hospital_2	M	79	53240	1	2	0	1
Chart_45	Hospital_2	F	77	53230	1	0	0	1
Chart_46	Hospital_2	M	80	53200	1	0	0	1
Chart_47	Hospital_2	M	80	53200	1	2	0	1
Chart_48	Hospital_2	F	83	53210	2	1	1	1
Chart_49	Hospital_2	F	87	53200	1	0	0	1
Chart_50	Hospital_2	M	73	53200	1	0	0	1
Chart_51	Hospital_2	M	66	53231	1	1	0	1
Chart_52	Hospital_2	M	79	53200	1	0	0	1
Chart_53	Hospital_2	M	85	53240	1	1	0	1
Chart_54	Hospital_2	M	27	53230	1	1	0	1
Chart_55	Hospital_2	M	53	53200	1	0	1	1
Chart_56	Hospital_2	F	91	53200	1	0	0	1
Chart_57	Hospital_2	M	30	53200	1	2	1	1
Chart_58	Hospital_2	M	64	53210	n.a.	n.a.	n.a.	n.a.

Number Clinical Chart	Hospital	Gender	Patient's age on admission	Primary Diagnosis (ICD-9)	Gastroscopy positive	Other positive instrumental test	Surgery positive	Validation
Chart_59	Hospital_2	F	53	53230	1	1	0	1
Chart_60	Hospital_2	F	84	53200	1	0	0	1
Chart_61	Hospital_2	M	76	53230	1	0	0	1
Chart_62	Hospital_2	M	76	53200	n.a.	n.a.	n.a.	n.a.
Chart_63	Hospital_2	M	62	53200	1	0	0	1
Chart_64	Hospital_2	F	82	53200	1	0	0	1
Chart_65	Hospital_2	M	80	53200	1	0	0	1
Chart_66	Hospital_2	F	75	53210	2	2	1	1
Chart_67	Hospital_2	M	86	53220	1	1	0	1
Chart_68	Hospital_3	M	43	53200	1	2	0	1
Chart_69	Hospital_3	M	77	53200	1	2	0	1
Chart_70	Hospital_3	F	82	53231	1	2	0	1
Chart_71	Hospital_3	M	84	53290	1	2	0	1
Chart_72	Hospital_3	F	86	53200	1	2	0	1
Chart_73	Hospital_3	M	77	53220	2	1	1	1
Chart_74	Hospital_3	M	68	53200	1	2	0	1
Chart_75	Hospital_3	M	89	53200	1	2	0	1
Chart_76	Hospital_4	M	59	53200	1	2	0	1
Chart_77	Hospital_4	F	90	53230	1	2	0	1
Chart_78	Hospital_4	F	80	53230	1	1	0	1
Chart_79	Hospital_4	M	62	53200	1	2	0	1
Chart_80	Hospital_4	M	68	53290	1	1	0	1
Chart_81	Hospital_4	F	55	53230	1	0	0	1
Chart_82	Hospital_4	M	91	53200	1	0	0	1
Chart_83	Hospital_4	F	47	53200	1	2	0	1
Chart_84	Hospital_4	M	52	53210	2	2	1	1
Chart_85	Hospital_4	F	88	53200	1	2	0	1
Chart_86	Hospital_4	M	72	53221	1	0	1	1
Chart_87	Hospital_4	M	45	53200	1	2	0	1
Chart_88	Hospital_4	F	59	53200	1	0	0	1

Number Clinical Chart	Hospital	Gender	Patient's age on admission	Primary Diagnosis (ICD-9)	Gastroscopy positive	Other positive instrumental test	Surgery positive	Validation
Chart_89	Hospital_5	M	70	53200	1	2	0	1
Chart_90	Hospital_5	F	55	53210	2	2	1	1
Chart_91	Hospital_5	F	63	53241	1	2	1	1
Chart_92	Hospital_5	M	20	53210	2	0	1	1
Chart_93	Hospital_5	M	39	53240	1	2	0	1
Chart_94	Hospital_5	M	73	53250	2	1	1	1
Chart_95	Hospital_5	M	69	53230	1	1	0	1
Chart_96	Hospital_5	F	88	53200	1	0	0	1
Chart_97	Hospital_5	M	73	53200	1	2	0	1
Chart_98	Hospital_5	M	78	53240	1	2	0	1
Chart_99	Hospital_5	M	79	53200	1	0	0	1
Chart_100	Hospital_5	M	83	53240	1	2	0	1
Chart_101	Hospital_5	F	70	53200	1	2	0	1
Chart_102	Hospital_5	F	83	53200	1	2	0	1
Chart_103	Hospital_5	M	77	53260	0	0	1	1
Chart_104	Hospital_5	F	55	53210	1	2	0	1
Chart_105	Hospital_5	M	58	53250	2	1	1	1
Chart_106	Hospital_5	F	90	53200	1	2	0	1
Chart_107	Hospital_5	M	78	53270	1	2	0	1
Chart_108	Hospital_5	M	59	53240	1	1	0	1
Chart_109	Hospital_5	M	80	53240	1	2	0	1
Chart_110	Hospital_5	M	58	53200	1	0	0	1
Chart_111	Hospital_5	M	87	53250	2	1	1	1
Chart_112	Hospital_6	F	57	53200	1	1	0	1
Chart_113	Hospital_6	F	95	53200	1	0	0	1
Chart_114	Hospital_6	M	47	53290	0	2	0	0
Chart_115	Hospital_6	M	71	53200	1	1	0	1
Chart_116	Hospital_6	F	90	53200	1	2	0	1
Chart_117	Hospital_6	M	93	53200	1	2	0	1
Chart_118	Hospital_6	F	68	53230	1	0	0	1

Number Clinical Chart	Hospital	Gender	Patient's age on admission	Primary Diagnosis (ICD-9)	Gastroscopy positive	Other positive instrumental test	Surgery positive	Validation
Chart_119	Hospital_6	F	60	53200	1	2	0	1
Chart_120	Hospital_6	F	87	53290	0	2	0	0
Chart_121	Hospital_6	F	65	53200	1	2	0	1
Chart_122	Hospital_6	M	85	53230	1	2	0	1
Chart_123	Hospital_6	M	85	53200	1	2	0	1
Chart_124	Hospital_7	M	61	53250	2	1	1	1
Chart_125	Hospital_7	M	67	53200	1	0	0	1
Chart_126	Hospital_7	F	69	53200	1	2	0	1
Chart_127	Hospital_7	F	58	53200	1	2	0	1
Chart_128	Hospital_7	F	75	53200	1	2	0	1
Chart_129	Hospital_7	M	82	53200	1	2	0	1
Chart_130	Hospital_7	M	46	53240	1	2	0	1

Legend: 0=no; 1=yes; 2=not reported; n.a.=clinical chart not available

Gastrojejunal ulcer

Number Clinical Chart	Hospital	Gender	Patient's age on admission	Primary Diagnosis (ICD-9)	Gastroscopy positive	Other positive instrumental test	Surgery positive	Validation
Chart_1	Hospital_1	M	53	53400	1	0	0	1
Chart_2	Hospital_1	M	74	53401	2	0	0	0
Chart_3	Hospital_1	M	69	53400	2	0	0	1
Chart_4	Hospital_1	M	70	53400	1	2	0	1
Chart_5	Hospital_1	M	69	53441	0	0	0	0
Chart_6	Hospital_1	M	86	53400	1	2	0	1
Chart_7	Hospital_1	M	66	53400	1	0	0	1
Chart_8	Hospital_2	F	82	53400	1	2	1	1
Chart_9	Hospital_2	F	71	53400	1	0	0	1
Chart_10	Hospital_2	F	88	53400	n.a.	n.a.	n.a.	n.a.
Chart_11	Hospital_2	M	77	53400	1	2	0	1
Chart_12	Hospital_2	F	88	53400	1	0	0	1
Chart_13	Hospital_2	F	85	53430	1	1	0	1
Chart_14	Hospital_2	F	53	53410	2	1	1	1
Chart_15	Hospital_3	M	63	53490	1	0	0	1
Chart_16	Hospital_3	M	77	53490	1	0	0	1
Chart_17	Hospital_4	M	51	53400	0	2	0	0
Chart_18	Hospital_4	M	73	53400	0	2	0	0
Chart_19	Hospital_4	M	79	53400	1	2	0	1
Chart_20	Hospital_4	F	92	53430	0	1	0	0
Chart_21	Hospital_4	M	84	53400	0	0	0	0
Chart_22	Hospital_4	M	82	53401	0	0	0	0
Chart_23	Hospital_4	F	69	53400	0	2	0	0
Chart_24	Hospital_4	M	63	53400	0	2	0	0
Chart_25	Hospital_4	F	93	53400	0	2	0	0
Chart_26	Hospital_4	M	75	53400	0	2	0	0
Chart_27	Hospital_4	M	81	53441	0	2	0	0

Number Clinical Chart	Hospital	Gender	Patient's age on admission	Primary Diagnosis (ICD-9)	Gastroscopy positive	Other positive instrumental test	Surgery positive	Validation
Chart_28	Hospital_4	M	75	53430	1	0	0	1
Chart_29	Hospital_4	M	79	53400	0	2	0	0
Chart_30	Hospital_4	M	74	53400	0	2	0	0
Chart_31	Hospital_4	M	87	53400	0	0	0	0
Chart_32	Hospital_4	F	84	53400	0	2	0	0
Chart_33	Hospital_4	M	75	53470	0	0	0	0
Chart_34	Hospital_4	F	91	53400	0	2	0	0
Chart_35	Hospital_4	F	76	53400	1	2	0	1
Chart_36	Hospital_4	M	68	53400	0	2	0	0
Chart_37	Hospital_4	F	88	53400	0	0	0	0
Chart_38	Hospital_5	F	81	53400	0	2	0	0
Chart_39	Hospital_5	F	88	53400	0	2	0	0
Chart_40	Hospital_5	F	77	53400	0	0	0	0
Chart_41	Hospital_5	F	87	53400	1	0	1	1
Chart_42	Hospital_5	M	93	53400	1	2	0	1
Chart_43	Hospital_5	M	90	53400	0	2	0	0
Chart_44	Hospital_5	M	28	53400	0	2	0	0
Chart_45	Hospital_6	F	88	53400	1	0	0	1
Chart_46	Hospital_6	F	87	53461	1	1	0	1
Chart_47	Hospital_6	F	60	53400	0	2	0	0
Chart_48	Hospital_6	M	22	53400	1	2	0	1
Chart_49	Hospital_6	F	81	53400	1	2	0	1
Chart_50	Hospital_6	F	39	53400	0	2	0	0
Chart_51	Hospital_6	M	84	53400	1	2	0	1
Chart_52	Hospital_6	F	89	53460	0	0	1	0
Chart_53	Hospital_6	M	76	53400	1	2	0	1
Chart_54	Hospital_6	F	75	53400	0	2	0	0
Chart_55	Hospital_6	F	81	53401	2	0	0	0
Chart_56	Hospital_7	F	84	53400	0	2	0	0
Chart_57	Hospital_7	M	72	53400	0	2	0	0

Number Clinical Chart	Hospital	Gender	Patient's age on admission	Primary Diagnosis (ICD-9)	Gastroscopy positive	Other positive instrumental test	Surgery positive	Validation
Chart_58	Hospital_7	M	72	53400	0	2	0	0
Chart_59	Hospital_7	M	58	53440	1	2	0	1
Chart_60	Hospital_7	F	86	53400	0	2	0	0
Chart_61	Hospital_7	F	37	53410	2	1	1	1
Chart_62	Hospital_7	F	93	53410	2	1	1	1
Chart_63	Hospital_7	M	86	53410	2	1	1	1

Legend: 0=no; 1=yes; 2=not reported; n.a.=clinical chart not available

Gastrointestinal hemorrhage

Number Clinical Chart	Hospital	Gender	Patient's age on admission	Primary Diagnosis (ICD-9)	Presence of anamnestic signs of bleeding	Gastroscopy positive	Colonscopy positive	Other positive instrumental test	Validation
Chart_1	Hospital_1	M	76	5781	1	1	2	2	1
Chart_2	Hospital_1	F	89	5781	1	2	2	2	1
Chart_3	Hospital_1	M	63	5781	1	2	2	2	1
Chart_4	Hospital_1	M	68	5781	1	2	1	2	1
Chart_5	Hospital_1	F	75	5789	0	2	2	1	1
Chart_6	Hospital_1	M	74	5781	1	0	1	1	1
Chart_7	Hospital_1	F	52	5789	1	2	2	0	1
Chart_8	Hospital_1	F	73	5789	0	2	1	0	1
Chart_9	Hospital_1	F	71	5781	1	0	1	2	1
Chart_10	Hospital_1	M	90	5789	0	0	1	0	1
Chart_11	Hospital_1	M	78	5789	1	2	1	2	1
Chart_12	Hospital_1	M	79	5781	1	2	1	1	1
Chart_13	Hospital_1	F	84	5781	1	0	1	2	1
Chart_14	Hospital_1	F	81	5789	1	1	0	2	1
Chart_15	Hospital_1	M	72	5780	1	1	2	2	1
Chart_16	Hospital_1	M	79	5780	1	1	2	2	1
Chart_17	Hospital_1	M	93	5781	1	0	1	2	1
Chart_18	Hospital_1	M	40	5781	1	1	2	2	1
Chart_19	Hospital_1	M	81	5781	1	2	1	2	1
Chart_20	Hospital_1	M	79	5789	1	1	1	2	1
Chart_21	Hospital_1	F	84	5789	1	0	1	2	1
Chart_22	Hospital_1	F	74	5789	0	1	1	1	1
Chart_23	Hospital_1	M	68	5789	0	1	2	2	0
Chart_24	Hospital_1	M	78	5781	1	2	2	2	1
Chart_25	Hospital_1	M	68	5780	1	1	2	0	1
Chart_26	Hospital_1	M	66	5781	1	2	1	2	1
Chart_27	Hospital_1	F	98	5781	1	2	2	1	1
Chart_28	Hospital_1	M	76	5780	1	1	2	0	1

Number Clinical Chart	Hospital	Gender	Patient's age on admission	Primary Diagnosis (ICD-9)	Presence of anamnestic signs of bleeding	Gastroscopy positive	Colonscopy positive	Other positive instrumental test	Validation
Chart_29	Hospital_1	F	79	5780	1	1	2	1	1
Chart_30	Hospital_1	M	#RIF!	5781	1	2	2	1	1
Chart_31	Hospital_1	F	94	5781	0	2	2	0	0
Chart_32	Hospital_1	M	40	5781	1	1	1	2	1
Chart_33	Hospital_1	F	71	5780	1	1	2	2	1
Chart_34	Hospital_1	M	60	5789	1	2	1	1	1
Chart_35	Hospital_1	F	91	5780	1	1	2	2	1
Chart_36	Hospital_1	M	64	5781	1	2	1	2	1
Chart_37	Hospital_1	F	90	5781	1	1	2	2	1
Chart_38	Hospital_1	F	67	5781	1	2	1	1	1
Chart_39	Hospital_1	M	79	5780	1	1	2	2	1
Chart_40	Hospital_1	M	88	5781	1	0	1	0	1
Chart_41	Hospital_1	F	77	5781	1	2	1	2	1
Chart_42	Hospital_1	F	83	5781	1	1	2	2	1
Chart_43	Hospital_1	M	87	5789	1	2	2	0	1
Chart_44	Hospital_1	F	89	5781	0	1	2	0	1
Chart_45	Hospital_1	F	74	5789	1	2	1	2	1
Chart_46	Hospital_1	F	51	5789	1	0	2	0	1
Chart_47	Hospital_1	M	39	5781	1	1	2	2	1
Chart_48	Hospital_1	F	80	5781	1	2	1	2	1
Chart_49	Hospital_1	F	75	5789	n.a.	n.a.	n.a.	n.a.	n.a.
Chart_50	Hospital_1	M	30	5781	1	2	1	2	1
Chart_51	Hospital_1	F	88	5780	1	2	2	2	1
Chart_52	Hospital_1	F	72	5780	n.a.	n.a.	n.a.	n.a.	n.a.
Chart_53	Hospital_1	F	90	5781	1	1	2	2	1
Chart_54	Hospital_2	F	100	5781	1	2	2	0	1
Chart_55	Hospital_2	F	41	5781	n.a.	n.a.	n.a.	n.a.	n.a.
Chart_56	Hospital_2	M	95	5781	1	2	2	2	1
Chart_57	Hospital_2	M	85	5780	1	0	2	0	1
Chart_58	Hospital_2	M	81	5781	1	2	2	1	1

Number Clinical Chart	Hospital	Gender	Patient's age on admission	Primary Diagnosis (ICD-9)	Presence of anamnestic signs of bleeding	Gastroscopy positive	Colonscopy positive	Other positive instrumental test	Validation
Chart_59	Hospital_2	F	97	5781	1	2	2	2	1
Chart_60	Hospital_2	M	89	5781	1	2	2	2	1
Chart_61	Hospital_2	M	76	5781	1	2	1	2	1
Chart_62	Hospital_2	F	80	5781	1	2	1	2	1
Chart_63	Hospital_2	M	50	5781	1	2	2	2	1
Chart_64	Hospital_2	F	82	5781	1	0	1	2	1
Chart_65	Hospital_2	M	65	5781	1	2	1	2	1
Chart_66	Hospital_2	F	82	5781	1	2	1	1	1
Chart_67	Hospital_2	M	41	5781	1	0	1	2	1
Chart_68	Hospital_2	F	94	5781	1	2	2	2	1
Chart_69	Hospital_2	M	89	5781	1	0	1	1	1
Chart_70	Hospital_2	F	88	5789	1	2	1	2	1
Chart_71	Hospital_2	F	65	5789	1	1	0	0	1
Chart_72	Hospital_3	F	65	5789	1	2	2	2	1
Chart_73	Hospital_3	F	83	5781	1	2	1	2	1
Chart_74	Hospital_3	M	81	5781	1	2	2	1	1
Chart_75	Hospital_3	M	73	5789	1	1	2	2	0
Chart_76	Hospital_3	M	86	5789	1	1	2	0	1
Chart_77	Hospital_3	M	66	5781	1	0	1	2	1
Chart_78	Hospital_3	F	67	5781	1	0	1	2	1
Chart_79	Hospital_3	M	36	5781	1	2	1	2	1
Chart_80	Hospital_3	F	88	5789	1	2	1	2	1
Chart_81	Hospital_3	M	79	5789	1	2	1	1	1
Chart_82	Hospital_4	F	81	5789	1	0	1	0	1
Chart_83	Hospital_4	M	88	5781	1	2	2	2	1
Chart_84	Hospital_4	M	73	5789	1	0	1	2	1
Chart_85	Hospital_4	F	74	5789	1	0	2	2	1
Chart_86	Hospital_4	F	63	5781	1	2	1	2	1
Chart_87	Hospital_4	M	62	5789	1	2	2	1	1
Chart_88	Hospital_4	M	64	5789	0	2	2	1	1

Number Clinical Chart	Hospital	Gender	Patient's age on admission	Primary Diagnosis (ICD-9)	Presence of anamnestic signs of bleeding	Gastroscopy positive	Colonscopy positive	Other positive instrumental test	Validation
Chart_89	Hospital_4	M	84	5789	1	1	1	2	1
Chart_90	Hospital_4	M	66	5789	1	2	2	1	1
Chart_91	Hospital_4	M	83	5789	1	2	1	2	1
Chart_92	Hospital_5	F	92	5789	1	1	2	2	1
Chart_93	Hospital_5	M	61	5789	1	0	1	2	1
Chart_94	Hospital_5	F	71	5780	1	2	2	0	1
Chart_95	Hospital_5	M	58	5789	1	2	2	1	1
Chart_96	Hospital_5	F	90	5781	1	2	2	2	1
Chart_97	Hospital_5	F	74	5789	1	0	1	2	1
Chart_98	Hospital_5	F	50	5781	1	2	0	0	1
Chart_99	Hospital_5	M	64	5781	1	2	1	1	1
Chart_100	Hospital_5	M	73	5789	1	1	2	2	0
Chart_101	Hospital_5	M	63	5781	1	2	1	2	1
Chart_102	Hospital_5	F	73	5780	1	2	2	1	1
Chart_103	Hospital_5	F	92	5781	1	2	1	2	1
Chart_104	Hospital_5	M	78	5789	1	1	2	2	0
Chart_105	Hospital_5	F	87	5781	1	2	1	2	1
Chart_106	Hospital_5	M	93	5781	1	2	1	2	1
Chart_107	Hospital_5	F	75	5789	1	1	1	2	1
Chart_108	Hospital_5	M	76	5789	0	2	1	2	1
Chart_109	Hospital_5	F	81	5781	1	2	2	2	1
Chart_110	Hospital_5	M	94	5789	1	1	2	1	1
Chart_111	Hospital_5	M	76	5781	1	2	1	2	1
Chart_112	Hospital_5	F	86	5781	0	1	2	2	0
Chart_113	Hospital_5	F	76	5781	1	1	2	0	1
Chart_114	Hospital_5	F	86	5781	1	2	1	2	1
Chart_115	Hospital_5	M	76	5781	0	1	2	0	0
Chart_116	Hospital_5	F	75	5781	1	2	1	1	1
Chart_117	Hospital_6	F	81	5781	1	1	2	0	1
Chart_118	Hospital_6	M	66	5789	1	1	0	1	1

Number Clinical Chart	Hospital	Gender	Patient's age on admission	Primary Diagnosis (ICD-9)	Presence of anamnestic signs of bleeding	Gastroscopy positive	Colonscopy positive	Other positive instrumental test	Validation
Chart_119	Hospital_6	F	85	5789	1	0	1	2	1
Chart_120	Hospital_6	M	88	5789	1	0	0	0	1
Chart_121	Hospital_7	F	81	5781	1	2	1	2	1
Chart_122	Hospital_7	M	45	5780	1	0	2	2	1
Chart_123	Hospital_7	M	46	5781	1	2	2	1	1
Chart_124	Hospital_7	M	77	5781	1	1	1	0	1
Chart_125	Hospital_7	F	25	5781	1	2	2	0	1
Chart_126	Hospital_7	F	85	5789	0	2	2	2	0
Chart_127	Hospital_7	M	74	5789	1	2	2	1	1
Chart_128	Hospital_7	F	86	5789	1	1	2	0	1
Chart_129	Hospital_7	F	88	5781	1	2	2	1	1
Chart_130	Hospital_7	F	93	5781	1	2	2	1	1

Legend: 0=no; 1=yes; 2=not reported; n.a.=clinical chart not available

Non-cases

Number Clinical Chart	Hospital	Gender	Patient's age at admission	Primary Diagnosis (ICD-9)	Non-cases group for 531.x - Validation	Non-cases group for 532.x - Validation	Non-cases group for 534.x - Validation	Non-cases group for 578.x - Validation
Chart_1	Hospital_1	M	76	41071	1	1	1	1
Chart_2	Hospital_1	F	79	1513	1	1	1	1
Chart_3	Hospital_1	F	78	56985	1	1	1	1
Chart_4	Hospital_1	F	22	53540	1	1	1	1
Chart_5	Hospital_1	F	73	5523	1	1	1	1
Chart_6	Hospital_1	M	38	5589	1	1	1	1
Chart_7	Hospital_1	M	40	53011	1	1	1	1
Chart_8	Hospital_1	F	62	56213	1	1	1	0
Chart_9	Hospital_1	F	41	57420	1	1	1	1
Chart_10	Hospital_1	F	74	5579	1	1	1	0
Chart_11	Hospital_1	M	75	59381	1	1	1	1
Chart_12	Hospital_1	F	27	0088	1	1	1	1
Chart_13	Hospital_1	M	55	5761	1	1	1	1
Chart_14	Hospital_1	M	46	07032	1	1	1	1
Chart_15	Hospital_1	M	79	1536	1	1	1	1
Chart_16	Hospital_1	M	37	0701	1	1	1	1
Chart_17	Hospital_1	M	85	57512	1	1	1	1
Chart_18	Hospital_1	F	79	57400	1	1	1	1
Chart_19	Hospital_1	M	75	5939	1	1	1	1
Chart_20	Hospital_1	F	61	56213	1	1	1	0
Chart_21	Hospital_1	M	71	1536	1	1	1	1
Chart_22	Hospital_1	F	50	57420	1	1	1	1
Chart_23	Hospital_1	F	76	1520	1	1	1	1
Chart_24	Hospital_1	F	25	0091	1	1	1	1
Chart_25	Hospital_1	F	82	1571	1	1	1	1
Chart_26	Hospital_1	F	84	5589	1	1	1	1
Chart_27	Hospital_1	F	70	56039	1	1	1	1

Number Clinical Chart	Hospital	Gender	Patient's age at admission	Primary Diagnosis (ICD-9)	Non-cases group for 531.x - Validation	Non-cases group for 532.x - Validation	Non-cases group for 534.x - Validation	Non-cases group for 578.x - Validation
Chart_28	Hospital_1	F	49	5589	1	1	1	1
Chart_29	Hospital_1	F	84	53783	1	1	1	1
Chart_30	Hospital_1	M	70	57450	1	1	1	1
Chart_31	Hospital_1	F	64	1536	1	1	1	1
Chart_32	Hospital_1	F	74	1977	1	1	1	1
Chart_33	Hospital_1	F	81	57420	1	1	1	1
Chart_34	Hospital_1	F	63	53649	1	1	1	1
Chart_35	Hospital_1	M	65	2809	1	1	1	1
Chart_36	Hospital_2	F	69	57410	1	1	1	1
Chart_37	Hospital_2	F	36	57410	1	1	1	1
Chart_38	Hospital_2	M	18	5368	1	1	1	1
Chart_39	Hospital_2	M	32	78909	1	1	1	1
Chart_40	Hospital_2	M	52	5528	1	1	1	1
Chart_41	Hospital_2	F	25	78900	1	1	1	1
Chart_42	Hospital_2	F	57	57410	1	1	1	1
Chart_43	Hospital_2	F	78	5565	1	1	1	1
Chart_44	Hospital_2	F	92	5602	1	1	1	1
Chart_45	Hospital_2	M	90	2164	1	1	1	1
Chart_46	Hospital_2	F	53	1529	1	1	1	1
Chart_47	Hospital_2	F	23	5770	1	1	1	1
Chart_48	Hospital_2	F	83	1541	1	1	1	1
Chart_49	Hospital_2	M	79	56211	1	1	1	1
Chart_50	Hospital_2	M	49	55090	1	1	1	1
Chart_51	Hospital_2	F	55	56211	1	1	1	1
Chart_52	Hospital_2	F	79	99591	1	1	1	1
Chart_53	Hospital_2	M	23	78900	1	1	1	1
Chart_54	Hospital_2	M	29	78903	1	1	1	1
Chart_55	Hospital_2	M	59	56983	1	1	1	1
Chart_56	Hospital_2	F	73	56213	1	1	1	1

Number Clinical Chart	Hospital	Gender	Patient's age at admission	Primary Diagnosis (ICD-9)	Non-cases group for 531.x - Validation	Non-cases group for 532.x - Validation	Non-cases group for 534.x - Validation	Non-cases group for 578.x - Validation
Chart_57	Hospital_2	F	78	1536	1	1	1	1
Chart_58	Hospital_2	F	82	5715	1	1	1	0
Chart_59	Hospital_2	M	36	6850	1	1	1	1
Chart_60	Hospital_2	M	67	1532	1	1	1	1
Chart_61	Hospital_2	F	42	57420	1	1	1	1
Chart_62	Hospital_2	M	31	5409	1	1	1	1
Chart_63	Hospital_2	F	28	566	1	1	1	1
Chart_64	Hospital_2	M	36	5756	1	1	1	1
Chart_65	Hospital_2	F	75	57420	1	1	1	1
Chart_66	Hospital_2	F	71	56409	1	1	1	1
Chart_67	Hospital_2	M	47	2638	1	1	1	1
Chart_68	Hospital_2	M	60	5738	1	1	1	1
Chart_69	Hospital_2	M	48	1550	1	1	1	1
Chart_70	Hospital_2	F	67	2113	1	1	1	1
Chart_71	Hospital_5	F	42	5300	1	1	1	1
Chart_72	Hospital_5	M	71	57451	1	1	1	1
Chart_73	Hospital_5	F	61	53081	1	1	1	1
Chart_74	Hospital_5	F	72	5690	0	1	1	1
Chart_75	Hospital_5	F	83	2851	0	1	1	0
Chart_76	Hospital_5	M	75	185	1	1	1	1
Chart_77	Hospital_5	M	58	1539	1	1	1	1
Chart_78	Hospital_5	M	75	2111	1	1	1	1
Chart_79	Hospital_5	M	63	2113	1	1	1	1
Chart_80	Hospital_5	M	48	53011	1	1	1	1

Legend: 0=no; 1=yes.
