# Supplementary material for: Accuracy of ICD-9 codes in identifying patients with peptic ulcer and gastrointestinal hemorrhage in the regional healthcare administrative database of Umbria
Source: PLoS One. 2020 Jul 6;15(7):e0235714. doi: 10.1371/journal.pone.0235714 (PMC7337287; doi:10.1371/journal.pone.0235714)
Supplement: Attachment — Submitted filename: Response to Reviewers.docx [file pone.0235714.s003.docx]

**PONE-D-20-09372: Response to Reviewers**

----- Messaggio inoltrato -----

Da: PLOS ONE <em@editorialmanager.com>

A: Iosief Abraha <iosief_a@yahoo.it>

Inviato: venerdì 15 maggio 2020, 23:00:05 CEST

Oggetto: PLOS ONE Decision: Revision required [PONE-D-20-09372] - [EMID:77a2dfc9439bf3c6]

PONE-D-20-09372

Accuracy of ICD-9 codes in identifying patients with peptic ulcer and gastrointestinal hemorrhage in the regional healthcare administrative database of Umbria.

PLOS ONE

Dear Dr. Abraha,

Thank you for submitting your manuscript to PLOS ONE. After careful consideration, we feel that it has merit but does not fully meet PLOS ONE’s publication criteria as it currently stands. Therefore, we invite you to submit a revised version of the manuscript that addresses the points raised during the review process.

The reviewers have provided useful comments to improve the manuscript.

We would appreciate receiving your revised manuscript by Jun 29 2020 11:59PM. When you are ready to submit your revision, log on to https://www.editorialmanager.com/pone/ and select the 'Submissions Needing Revision' folder to locate your manuscript file.

If you would like to make changes to your financial disclosure, please include your updated statement in your cover letter.

To enhance the reproducibility of your results, we recommend that if applicable you deposit your laboratory protocols in protocols.io, where a protocol can be assigned its own identifier (DOI) such that it can be cited independently in the future. For instructions see: http://journals.plos.org/plosone/s/submission-guidelines#loc-laboratory-protocols

Please include the following items when submitting your revised manuscript:

A rebuttal letter that responds to each point raised by the academic editor and reviewer(s). This letter should be uploaded as separate file and labeled 'Response to Reviewers'.

A marked-up copy of your manuscript that highlights changes made to the original version. This file should be uploaded as separate file and labeled 'Revised Manuscript with Track Changes'.

An unmarked version of your revised paper without tracked changes. This file should be uploaded as separate file and labeled 'Manuscript'.

Please note while forming your response, if your article is accepted, you may have the opportunity to make the peer review history publicly available. The record will include editor decision letters (with reviews) and your responses to reviewer comments. If eligible, we will contact you to opt in or out.

We look forward to receiving your revised manuscript.

Kind regards,

Gianni Virgili

Academic Editor

PLOS ONE

Journal Requirements:

When submitting your revision, we need you to address these additional requirements.

1. Please ensure that your manuscript meets PLOS ONE's style requirements, including those for file naming. The PLOS ONE style templates can be found at

https://journals.plos.org/plosone/s/file?id=wjVg/PLOSOne_formatting_sample_main_body.pdf and

https://journals.plos.org/plosone/s/file?id=ba62/PLOSOne_formatting_sample_title_authors_affiliations.pdf

[Note: HTML markup is below. Please do not edit.]

Reviewers' comments:

Reviewer's Responses to Questions

Comments to the Author

1. Is the manuscript technically sound, and do the data support the conclusions?

The manuscript must describe a technically sound piece of scientific research with data that supports the conclusions. Experiments must have been conducted rigorously, with appropriate controls, replication, and sample sizes. The conclusions must be drawn appropriately based on the data presented.

Reviewer #1: Yes

Reviewer #2: Yes

2. Has the statistical analysis been performed appropriately and rigorously?

Reviewer #1: Yes

Reviewer #2: Yes

3. Have the authors made all data underlying the findings in their manuscript fully available?

The PLOS Data policy requires authors to make all data underlying the findings described in their manuscript fully available without restriction, with rare exception (please refer to the Data Availability Statement in the manuscript PDF file). The data should be provided as part of the manuscript or its supporting information, or deposited to a public repository. For example, in addition to summary statistics, the data points behind means, medians and variance measures should be available. If there are restrictions on publicly sharing data—e.g. participant privacy or use of data from a third party—those must be specified.

Reviewer #1: No

Reviewer #2: Yes

4. Is the manuscript presented in an intelligible fashion and written in standard English?

PLOS ONE does not copyedit accepted manuscripts, so the language in submitted articles must be clear, correct, and unambiguous. Any typographical or grammatical errors should be corrected at revision, so please note any specific errors here.

Reviewer #1: Yes

Reviewer #2: Yes

5. Review Comments to the Author

Please use the space provided to explain your answers to the questions above. You may also include additional comments for the author, including concerns about dual publication, research ethics, or publication ethics. (Please upload your review as an attachment if it exceeds 20,000 characters)

**Reviewer #1**: In this study authors carried out a validation study by using the Regional Healthcare administrative database of Umbria. As mentioned in the manuscript authors have published other article aimed to validate cardiovascular and cerebrovascular diseases. The article is well written, understandable and well structured. Thank you for your positive comment.

However, I have some comments that authors should take into account:

1) In the method section, the authors declare that they used ICD9 included in the primary position of the hospital discharge database. I am aware that sometime in second position can be included only a few information, but I was wondering why authors did not include also this codes in the validation (i.e., at least for most important event such as gastric haemorrhage).

**Authors response**: We decided to limit our validation study to only consider the codes in primary position because, according to the Italian legislation, the primary diagnosis constitutes the main cause of the need for treatment and/or diagnostic tests, and is mainly responsible for the use of resources.

We added this sentence to the study limitations: “*We acknowledge that a potential limitation of our study is that we evaluated the accuracy of ICD-9 codes located only in primary position.* ***We chose to limit our analysis only to the codes in primary position because, according to the Italian legislation, the primary diagnosis constitutes the main cause of the need for treatment and/or diagnostic tests, and is mainly responsible for the use of resources****”*.

2) In the discussion authors declare that other studies have been performed in Italy with the same purpose. Can authors better specify the difference among the other two studies and their one? In addition, can authors specify what their paper adds to the Italian literature on this topic?

**Authors response**: The main difference between our study and the other two Italian studies on this topic is that we were interested in validating the complete ICD-9 codes 531, 532, 534, and 578, while Cattaruzzi et al. and Pisa et al. considered the same ICD-9 codes of our study (peptic ulcer and gastrointestinal hemorrhage), but limited to the sub-codes of hemorrhage or perforation, with the aim to identify patients with upper gastrointestinal bleeding (UGIB) associated with intake of nonsteroidal antiinflammatory drugs (NSAIDs) and other drugs.

We decided to perform our study in order to validate for the first time the Regional administrative database of Umbria for these specific codes, that could be used in future to perform other epidemiological studies and health services research.

We added this in the discussion section: “*The present study is one of the few in Italy* ***and the first in Umbria Region*** *validating the ICD-9 codes related to peptic ulcer and gastrointestinal hemorrhage using clinical charts as a reference standard*”.

3) I partly agree with authors’s sentence “….validation studies of administrative database are context-specific, and thus our results can be applied only to the regional setting of Umbria”. What about the use/adaptation of the same codes in other Italian region? Do authors suppose a different diagnosis procedures/codes used in other Italian regions? If yes, why?

**Authors response**: In general, results that originate from a healthcare database are immediately applicable only to the setting in which such database has been validated, due to differences that may exist with respect to demographics, disease prevalence, and standards of care, among different regions.

However, we think that our methodology could be replicated in other settings in order to identify possible differences in diagnostic accuracy results.

We amended the text as follows: “*Regarding the generalizability of our study, we want to highlight that, in general, validation studies of administrative databases are context-specific* ***due to differences that may exist in demographics, disease prevalence, and standards of care among different contexts****, and thus our results can* ***confidently*** *be applied only to the regional setting of Umbria*”.

**Reviewer #2**: Dear authors,

Your manuscript gives interesting informations on the accuracy of ICD-9 codes in identifying patients with peptic ulcer and gastrointestinal haemorrhage in the regional healthcare administrative database of Umbria and the validated codes will be useful for future epidemiological studies on health services.

Thank you for your positive comment.

However, there are certain points that need to be clarified or better discussed:

1) In the "setting and datasource paragraph", no information on medical charts were presented. Please explain the origin of these medical charts. From which hospital did you retrive these data? Is there a data warehouse collecting these informations?

**Authors response**: We added in the text the description of the hospitals where the clinical charts came from: “*We considered all the residents in the Umbria Region > 18 years discharged* ***from seven hospitals (Perugia, Terni, Foligno, Città di Castello, Orvieto, Gubbio-Gualdo Tadino, Spoleto)*** *between 2012 and 2014 with a diagnosis of peptic ulcer or gastrointestinal haemorrhage*”.

We presented a minimal anonymized dataset containing the main characteristics of the study sample (see Supplementary material).

2) Despite the great effort in analysing sensitivity and specificity of ICD-9codes, the authors measure those values only on the percentage of 80 “non cases”. Do you know the “real prevalence” of patients with these diseases in the Umbria region between 2012 and 2014? Is there a pathology registry in Umbria to compare the prevalence of diseases found using administrative data with the “real prevalence” of these diseases in an already validated registry? If these data cannot be recovered (or are inexistent) please discuss in the discussion section.

**Authors response**: Unfortunately, in Umbria there is not a pathology registry for ulcers or gastrointestinal hemorrhage, and therefore we do not know the real prevalence of the diseases. We based our estimation of sensitivity and specificity using a sample of “non-cases”, i.e. patients who had been discharged in the same period in a gastroenterology ward with a diagnosis other than peptic ulcer or gastrointestinal hemorrhage, from which we extracted a random sample of 80 patients.

We added in the discussion section the following sentence: “*Instead, in order to estimate sensitivity and specificity, in absence of a disease registry for peptic ulcers and gastrointestinal hemorrhage that constitutes the real prevalence of the diseases, we chose to consider a sample of “non-cases”, i.e. patients who had been discharged in the same period in a gastroenterology ward with a diagnosis other than peptic ulcer or gastrointestinal hemorrhage, to individuate possible false negatives*”.

3) Have you performed any sensitivity analyses considering not only ICD-9 CM codes in the primary position but also using the secondary positions? If not, please discuss further the motivations in using only primary position codes in the limitations of the study.

**Authors response**: We did not perform a sensitivity analysis considering the codes in secondary position because all our study sample was discharged with a diagnosis code in primary position (we did not extracted patients with the codes in secondary positions).

We explained in the limitations the reasons why we chose to focus on the codes in primary position, adding the following sentence: “*We chose to limit our analysis only to the codes in primary position because, according to the Italian legislation, the primary diagnosis constitutes the main cause of the need for treatment and/or diagnostic tests, and is mainly responsible for the use of resources*”.

4) Please define abbreviations upon first appearance in the text. PPV and NPV abbreviation was firstly used in the "statistical analysis” section but the explanation is reported more than once in the “results” section.

**Authors response**: Thank you for your suggestion. We amended the text accordingly.

6. PLOS authors have the option to publish the peer review history of their article (what does this mean?). If published, this will include your full peer review and any attached files.

If you choose “no”, your identity will remain anonymous but your review may still be made public.

Do you want your identity to be public for this peer review? For information about this choice, including consent withdrawal, please see our Privacy Policy.

Reviewer #1: No

Reviewer #2: Yes: Andrea Spini

[NOTE: If reviewer comments were submitted as an attachment file, they will be attached to this email and accessible via the submission site. Please log into your account, locate the manuscript record, and check for the action link "View Attachments". If this link does not appear, there are no attachment files to be viewed.]

While revising your submission, please upload your figure files to the Preflight Analysis and Conversion Engine (PACE) digital diagnostic tool, https://pacev2.apexcovantage.com/. PACE helps ensure that figures meet PLOS requirements. To use PACE, you must first register as a user. Registration is free. Then, login and navigate to the UPLOAD tab, where you will find detailed instructions on how to use the tool. If you encounter any issues or have any questions when using PACE, please email us at figures@plos.org. Please note that Supporting Information files do not need this step.

In compliance with data protection regulations, you may request that we remove your personal registration details at any time. (Remove my information/details). Please contact the publication office if you have any questions.
